# Microdecompression for lumbar synovial cysts: an independent assessment of long term outcomes

**DOI:** 10.1186/1749-799X-2-5

**Published:** 2007-04-03

**Authors:** Bradley K Weiner, Joel Torretti, Michael Stauff

**Affiliations:** 1 Division of Spinal Surgery The Methodist Hospital 6550 Fannin, Suite 2500 Houston, Texas 77030, USA; 2 Department of Orthopaedics Dartmouth Hitchcock Medical Center Hanover, New Hampshire, USA; 3 Penn State College of Medicine Hershey, Pennsylvania, USA

## Abstract

**Background:**

Outcomes of surgical intervention for lumbar synovial cysts have been evaluated in the short and intermediate term. Concerns regarding cyst recurrence, the development of late instability at the involved level, and instability/stenosis at adjacent levels (when concomitant) fusion is performed suggest that long term follow-up is needed. This study aims to fill that void.

**Methods:**

Forty-six patients operated by a single surgeon not involved in the study were followed up long term at an average of 9.7 years (range 5 to 22 years) post-operatively. All patients underwent decompression (+/- concomitant arthrodesis in the presence of associated degenerative spondylolisthesis) using the operative microscope for magnification/illumination. Outcomes were assessed using a customized questionnaire evaluating: relief of pain/claudicant symptoms, numbness/parasthesias, and weakness; as well as late onset low back pain, new radicular symptoms, need for additional surgery, and patient satisfaction. Outcomes in patients with or without fusion were compared as well.

**Results:**

87% of patients noted resolution of their pre-operative pain, numbness, and weakness. 28% of patients developed late onset low back pain. 17% developed late onset radicular symptoms in a new nerve root distribution. 15% required subsequent additional surgery. 89% of patients were satisfied with the surgical outcome. No differences were found for any outcome measure between patients undergoing concomitant fusion and those undergoing decompression alone using the two-sample t-test.

**Conclusion:**

This study provides outcome data at an average of nearly ten years post-operative. This information should allow surgeons to provide realistic expectations for their patients regarding outcomes and should enhance the informed consent and surgical decision-making process.

## Background

**Figure 1:**
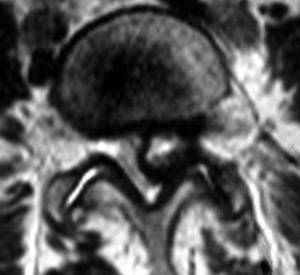
A typical case of synovial cyst at L5-S1.

Although originally recognized in peripheral joints by Baker in 1877[1,2], synovial cysts of the lumbar facet joints were not described until 1950 in the German literature [3,4] and were first well-delineated in English by Kao in the late 1960's/early 1970's [5,6]. Since then, CT and MRI scanning (Figure 1) have afforded highly sensitive and specific diagnosis of the cysts and the oft-associated compression of neurological structures. Such compression can result in radiculopathy, neurogenic claudication, and, rarely, cauda equina syndrome [7-11].

While multiple non-operative therapies have been implemented [12-16], few have demonstrated significant or lasting efficacy when used to treat patients with moderate or severe symptoms such as intractable pain or neurological deficit[12,14,16,17]. Accordingly, surgical intervention is commonly performed on patients in this group and several studies have demonstrated reasonable outcomes at short to intermediate term follow-up [7,11,15,18-23]. The longest follow-up published prior to the current study has been forty months and concerns about recurrence of the cysts, instability at the involved level (when isolated decompression is undertaken), or instability at adjacent levels (when concomitant fusion is performed) suggest that a longer-term look is needed to better understand the implications of our interventions.

The purpose of this study was to independently evaluate the long-term clinical outcomes in patients who underwent microdecompression with or without concomitant arthrodesis for symptomatic lumbar synovial cysts unresponsive to non-operative measures. The average follow-up of 9.7 years (range five to 22 years) represents the longest follow-up to date; the minimum follow-up for inclusion of five years being greater than the previously reported maximum follow-up of 3.25 years.

## Methods

### Surgical Technique

Patients were placed under general endotracheal anesthesia and placed in a kneeling position on a standard frame. The involved level(s) was marked preoperatively using c-arm imaging. A midline skin incision was made and the dorsolumbar fascia incised just lateral to the midline ipsilateral to the synovial cyst. Unilateral laminae were exposed using the Cobb elevator to the mid-portion of the facet joint. An intraoperative radiograph was used to confirm the level. A laminotomy on the undersurface of the cephalad lamina was undertaken to mirror the cephalad extent of the cyst as determined by pre-operative MRI. A similar caudal laminotomy was performed, again to mirror the extent of the cyst. Ligmantum flavum was then excised and the subarticular and foraminal zones decompressed via complete excision of soft-tissue/bony stenosing lesions to include extirpation of the synovial cyst. If the cyst was adherent to the dura (a common finding), it was carefully teased free so that no cyst pseudocapsule remained. The facet joint was opened and residual synovial tissue excised. If the patient had neurogenic claudication, a contralateral microdecompression as previously described[24] was undertaken. If the patient had an associated degenerative spondylolisthesis, bilateral uninstrumented intertransverse fusion as well as facet joint fusion was undertaken as previously described[25]. Magnification/illumination was provided by the operative microscope in all cases. The wound was irrigated, hemostasis obtained, and closure carried out in standard fashion.

### Patients (Table 1)

**Table 1:** Patient Characteristics

*Number*	*46*	
*Age in years*	*73 (range 25–96)*	
		
*Sex*	*29 Females 17 Males*	
		
*Anatomic Level of Cyst*	*L4-L5*	*28*
	*L5-S1*	*8*
	*L3-L4*	*6*
	*L2-L3*	*1*
	*L1-L2*	*1*
		
*Clinical Syndromes*	*Neurogenic Claudication*	*28*
	*Monoradiculopathy*	*18*
		
*Associated Degenerative Spondylolisthesis*		*23*

Forty-six patients operated between 1984 and 2001 were available for follow-up. All surgeries were performed by a single surgeon who was not involved in the study. Age at surgery ranged from 25 to 96 years with a mean of 73 years. Twenty-nine were females and seventeen males. Twenty-eight cysts were at the L4-L5 level, eight at L5-S1, six at L3-L4, and one each at L1-L2 and L2-L3. Clinical syndromes included unilateral monoradiculopathy in eighteen patients and neurogenic claudication in twenty-eight. Radiographically, twenty-three had an associated degenerative spondylolisthesis and underwent concomitant arthrodesis. This was the only indication for fusion in the study population. This follow-up study was approved by the institutional review board and oral consent was obtained from all participants.

### Data

Clinical outcomes and patient satisfaction were assessed by two independent spine surgeons using the questionnaire in Table 2. All forty-six patients responded.

**Table 2:** Questionnaire

*1. Do you have numbness or tingling in your leg(s) similar to what you had before surgery? (Better, Same, Worse)*
*2. Do you have weakness in your leg(s) similar to what you had before surgery? (Better, Same, Worse)*.
*3. Do you still have pain/symptoms in the same site that made you have surgery in the first place? (Rated on Visual Analog Scale)*
*4. Have you developed back pain over the years that is new/different than before surgery? (Rated on Visual Analog Scale)*
*5. Have you developed leg pain over the years that is new/different than before your surgery? (Rated on Visual Analog Scale)*
*6. Have you had additional surgery on your back? (Type of surgery, Reason for surgery)*
*7. Are you happy with the results of the surgery and would you recommend it to a friend with the same problem?*

*Positive responses on questionnaire were then followed up for specific details via telephone interview*.

## Results (Table 3)

**Table 3:** Summary of Results

***Complaint***	***% of patients having; % of patients symptom-free***
*Same Site Pain/Symptoms*	*12% averaging 5.5 on VAS; 88% resolved*
*Same Site Numbness/Tingling*	*9% same, 4% worse; 87% resolved*
*Same Site Weakness*	*11% same, 5% worse; 84% resolved*
*New Back Pain*	*28% averaging 7.5 on VAS; 72% pain free*
*New Radiculopathy*	*17% averaging 7.4 on VAS; 83% pain free*
*Additional Surgery*	*15%*

Follow-up averaged 9.7 years with a range of five to 22 years.

### Same-Site Pain/Similar Symptoms

Forty of the forty-six patients (88%) reported relief of their preoperative pain/symptoms. Six (12%) had persisting complaints ranked in severity at an average of 5.5 on the visual analog scale (VAS: range 2–10) versus an average of 9 on VAS preoperatively. All patients had pain or claudication preoperatively.

### Same-site Numbness

Twenty-three patients (50%) reported preoperative numbness. Of these, at follow-up, twenty (87%) reported complete or near-complete resolution, two (9%) were the same, and one (4%) was worse.

### Same-Site Weakness

Nineteen (41%) had complained of weakness prior to surgery. Of these, at follow-up, sixteen (84%) reported complete resolution, two (11%) reported no significant change in strength, and one (5%) was worse than preoperatively.

### New Back Pain

After initially doing well, thirteen patients (28%) reported the eventual development of new back pain ranked on average at 7.5 on the VAS.

### New Leg Pain

Eight patients (17%) reported the eventual onset of new radicular leg pain (different root involved) with a mean VAS severity of 7.4.

### Additional Surgery

Seven patients (15%) reported the need for additional lumbar spine surgery. Three patients who had not undergone fusion at the initial surgery required eventual revision decompression and fusion to include the operated levels due to instablility. Four patients who had undergone concomitant arthrodesis at the primary surgery due to presence of a degenerative spondylolisthesis eventually developed juxtafusional stenosis/instability requiring secondary decompression and fusion at involved adjacent levels.

### Patient Satisfaction

Forty-one patients (89%) reported overall satisfaction with the outcome of their initial procedure and would recommend it to a friend with the same problem.

### Did Presence of a Spondylolisthesis/Need for Fusion Alter Outcomes?

There were no statistically significant differences for *any *of the outcome measures above between patients presenting without a degenerative spondylolisthesis (decompression alone) and those presenting with one (decompression with concomitant fusion) using the two-sample t-test.

## Discussion and Conclusion

This study demonstrates that at an average of nearly ten years following decompressive surgery for symptomatic lumbar synovial cysts (with associated fusion if a degenerative spondylolisthesis is present), patients can anticipate: (a) about an 85% likelihood that their preoperative pain/claudication, numbness, and weakness will be resolved, (b) about a 25% likelihood of developing later onset back pain, (c) about 15% likelihood of developing later onset radicular symptoms in a new nerve root distribution, (d) that they have a 15% likelihood of needing additional lumbar surgery, and that (e) about nine out of ten patients are happy with the results of surgery.

These findings are generally commensurate with those of other studies having evaluated outcomes at much shorter intervals. Howington[7] achieved 88% good/excellent results at 40 months and Lyons[15] found similar results but with much shorter term follow-up. Khan[23] reported about 80% success rates at 26 months. At the extremes are Sandhu[20], Metellus[19], Pirotte[22], and Trummer[18] who reported between 95% and 100% success rates; and Epstein[21] who reported 60% good/excellent results at 24 months. The former studies likely representing snapshots commonly encountered in short-term retrospective studies, the latter probably representing cases associated with the need for more extensive laminectomies given that 16 of 66 patients in this study went onto develop significant and progressive instability at the operated level.

The value of the current study, given that its follow-up is dramatically longer than any previously published, is that it demonstrates, generally, that the beneficial effects of surgical intervention seen at shorter and intermediate time frames appear to persist, however some patients will develop late-onset low back pain, radicular pain, and may need additional surgery long term. This additional information should allow surgeons to provide realistic expectations for their patients regarding outcomes and should enhance the informed consent and surgical decision-making process.

The potential weaknesses of long term studies such as this are two-fold. First, over a period of ten to twenty years patients may go on to further degeneration or develop new medical comorbidities such that their overall health status (SF-36) or disease specific status (ODI) may actually appear worse than their pre-operative status – despite the fact that their specific reasons for surgery (e.g.; severe L5 root pain) may well have been relieved by the intervention. Long term studies are one of the rare cases where very specific outcome measures are indicated to ferret out this information, hence the choice of custom questionnaire used. Second, over that ten to twenty year period of time, the standards of care and the evidence base may have changed such that the information provided by the study is no longer relevant. This is a common problem in the total hip/knee replacement literature where long term outcomes are provided for prostheses no longer manufactured and surgical approaches no longer used. Just as the first potential weakness was avoided by intention, this second potential weakness was avoided by good fortune. Twenty-two years down the road, the standard of care, commensurate with the current evidence base, remains decompression of involved neurological tissue by complete excision of the cyst (including residual synovial tissue to avoid recurrence), excision of ligamentum flavum and other soft tissue and bony compressive pathology, and concomitant arthodesis in the presence of a degenerative spondylolisthesis.
